# Correction: Historical overview of the interleukin-6 family cytokine

**DOI:** 10.1084/jem.2019034704212020c

**Published:** 2020-04-27

**Authors:** Sujin Kang, Masashi Narazaki, Hozaifa Metwally, Tadamitsu Kishimoto

Vol. 217, No. 5, May 4, 2020. 10.1084/jem.20190347

The authors regret that an incorrect reference was cited in the original version of their paper. The reference and citation of Kishimoto, 1989 have been replaced with Sehgal et al., 1989. The revised sentence and complete reference appear below. All versions of the article have been corrected.

“The molecule was first designated IL-6 in 1988 at a conference entitled ‘Regulation of the Acute Phase and Immune Responses: A New Cytokine’ (Sehgal et al., 1989).”

Sehgal, P.B., G. Grieninger, and G. Tosato, editors. 1989. Regulation of the Acute Phase and Immune Responses: Interleukin-6. *Ann. N Y Acad. Sci.* 557:1–583.

